# Selected Behaviors and Addiction Risk Among Users of Urban Multimedia Games

**DOI:** 10.3389/fpsyg.2022.862891

**Published:** 2022-03-28

**Authors:** Mateusz Grajek, Łukasz Olszewski, Karolina Krupa-Kotara, Agnieszka Białek-Dratwa, Krzysztof Sas-Nowosielski

**Affiliations:** ^1^Department of Public Health, Faculty of Health Sciences, Medical University of Silesia in Katowice, Bytom, Poland; ^2^Department of Epidemiology and Biostatistics, Faculty of Health Sciences, Medical University of Silesia in Katowice, Bytom, Poland; ^3^Department of Dietetics, Faculty of Health Sciences, Medical University of Silesia in Katowice, Bytom, Poland; ^4^Department of Humanistic Foundations of Physical Culture, Academy of Physical Education in Katowice, Katowice, Poland

**Keywords:** physical activity, mental health, addiction to games, multimedia city games, Pokemon GO

## Abstract

**Introduction:**

The rapid development of technology has led to the transfer of entertainment to the virtual world. Many games and multimedia applications use the so-called augmented reality. With the development of a new technological branch, a new health problem has emerged, which is infoholic addiction, attracting people with the specific functionality that is cyberspace and the virtual world.

**Objective:**

The study aimed to assess health behaviors and the risk of addiction among users of urban multimedia games. Research methodology. The study was conducted among players 1,134 of urban multimedia games—Pokemon GO, in the period March–June 2021. A 990 correctly completed questionnaires were included in the final analyses. The author’s questionnaire was used in the study, which included two standardized measurement scales in the Polish abbreviated version: a questionnaire of emotions and sensations associated with electronic entertainment and a questionnaire on addiction to electronic forms of entertainment. Statistica 13.0 program was used for statistical processing of the data. The probability level was *p* < 0.05.

**Results:**

The profile of the gamer was identified as male, aged 18–30 years, with secondary education (more rarely higher), not in a relationship, living in a city of more than 100 thousand inhabitants—60.1% of gamers met these criteria. Respondents played urban multimedia games daily (84.7%). About 26.3% of respondents played this type of game 2–3 h a day. In terms of physical fitness, 64.3% of respondents felt that physical fitness had improved as a result of playing multimedia urban games. In terms of mental condition, most of the respondents said that nothing had changed (55.3%). It was observed that 72.3% of respondents had some risk of addiction to urban multimedia games.

**Conclusion:**

Users of urban multimedia games were usually adult men living in big cities. It was also shown that the defined profile of the player was characterized by a higher risk of addiction to electronic forms of entertainment. It was observed that the respondents most often played urban multimedia games every day in a maximum of two-hour sessions. Based on the tools used, it can be concluded that the study group was characterized by a certain risk of addiction to urban multimedia games.

## Introduction

In the age of affordable access to the latest technology, anyone can own an advanced device designed for work, entertainment, news, or various life tips. Nevertheless, more and more often we hear about the danger of mobile games addiction. There are new productions on the market and even smartphones dedicated to gamers. This makes that every day more people start to use mobile devices pathologically. As of 1 January 2022, gaming addiction has entered the WHO list of diseases (WHO, 2022). Gaming disorder, as the disorder has been named, includes limited control over games and prioritizing games. Special attention in estimating the risk of addiction to games should be paid to the so-called urban multimedia games, which are becoming increasingly popular not only among young people. These games, due to their off-road nature, are sometimes praised for their beneficial effects on the user’s health, but it is also worth considering the risk of addiction to this type of entertainment. Few of the current studies on urban multimedia games address their adverse effects. Available publications mainly focus on the physical activity aspect. Therefore, the research work described in this paper is focused on answering the question:

Do urban multimedia games influence health behavior and the risk of developing an addiction to these forms of entertainment among their users?

To answer the above question, the research work was divided into three stages:

First: Identify the audience for mobile urban games (in terms of gender and age, education, and location); Second: Assessing the impact that these types of games have on a player’s daily life (in terms of time played and impact on physical, mental, and interpersonal fitness); Third: Assessing the risk of addiction to urban multimedia games.

The following sections of the paper describe the research background—the context of the emergence and popularity of urban multimedia games, and the risk of multimedia addiction. The next section describes in detail the research material and methods used. The results section provides a synthesis of all the work conducted in the study and interprets the data obtained, including the results of the tools used to assess the risk of addiction to urban multimedia games. The results discussion section attempts to compare previous research findings with our research. The paper concludes with a section on the strengths and weaknesses of the study conducted and a substantive conclusion.

## Background

### About Multimedia Urban Games

The rapid development of technology has led to the transfer of entertainment to the virtual world. Many games and multimedia applications use the so-called augmented reality, which is the simultaneous existence of the real world with superimposed virtual elements. This trend did not bypass smartphone users and since the beginning of the second decade of the 2000s, we are flooded with applications whose authors outdo each other in functionality and convenience for everyday life. One of the types of such applications is mobile urban games such as Ingress, Pokemon GO, Draconius GO, Jurassic World Alive, and Harry Potter: Wizards Unite. The popularity of these types of games makes more and more people reach for them out of curiosity. An urban game is a form of play that is carried out in an urban space. It usually requires active movement, but also mental effort and sometimes sports. We can include all kinds of games such as stealth games, but also computer games that use augmented reality (Warcholik and Leja, 2012). One of the first of its kind was the game Pokemon GO, released by the company Niantic in July 2016, which combines the principles of a multimedia urban game with fictional creatures created in 1996 in Japan and spread around the world with the help of console games, card games, and animated movies. The game has been very well received worldwide, being the most used and profitable mobile app in 2016. By May 2018, it had more than 147 million active users and was the second product from Niantic after Ingress to combine terrain elements with augmented reality (Phillips, 2018).

Pokemon GO, as an urban game, requires the player to actively move around the area to catch virtual creatures and utilize other game mechanics. These also include tasks in the form of covering a given distance, for example, to hatch a “Pokemon egg” (2 km, 5 km, 7 km, or 10 km). The popularity of the game has attracted the interest of public health researchers, who have found both positive and negative aspects of using the app. In surveys, Pokemon GO users indicated an increase in individual physical activity, which at the same time was a good way to fight excessive body weight or obesity. Nevertheless, many news media outlets have reported on car accidents, injuries, trespassing in dangerous areas, or assaults through the Pokemon GO app (Barbero et al., 2018; Laor, 2021a). The popularity of Pokemon GO has allowed various developers to spread other urban multimedia games based on popular franchises including Harry Potter, Jurassic Park/World, and The Witche.

### About Addiction to Electronic Entertainment

Most addiction research addresses two primary factors in behavioral disorders: functional impairment and dysfunctional behavior. Of course, when comparing functional impairment in alcohol, cocaine, or heroin abuse with computer game addiction, a significant difference will emerge (Ducheneaut and Moore, 2004). The consequences of excessive gaming are less severe than psychoactive or narcotic substance use, although going another route nicotine addiction usually does not have significant consequences in family, work, or academic life unlike gaming addiction, although it does have much more harmful effects on the human body. Among the causes of addiction to computer games proposed by Fuster et al. (2012) and Young (1998) are the translation of the virtual world over the real one and identification with the avatar in the game. Massively multiplayer online role-playing games (MMORPGs) have the greatest potential for addiction because it is there that the user creates his character and gets to know the fictional world, which can also lead to ignoring and forgetting about the problems of the real world. Significant differences in the consequences of game abuse also depend on the game mechanics themselves (Ducheneaut and Moore, 2004; Seay et al., 2004). When playing games of the MMORPG genre, there will be different health consequences for a solitary and static person playing World of Warcraft at home, and a different for a dynamic person playing Pokemon GO on the street (Carbonell, 2017). The term Internet Addiction Disorder (IAD) was introduced in 2013 by the American Psychiatric Association. IAD has been defined as a persistent, recurrent, and excessive engagement in computer and video games that is not controlled despite co-existing problems. The term has also been identified as needing further research work, and its alignment with the term HGI is intended to help gather more information about it (Kevin and John, 2018).

With the development of the new technological branch, a new health problem has emerged, which is infoholic addiction, attracting people with the specific functionality that is cyberspace and the virtual world. The cell phone is now one of the most popular leisure activities, which dominates not only the areas of individual life but also cultural and social life. The problem of infoholic addiction, despite being recognized as serious by specialists, is still underestimated by the general public, especially because its effects do not bring as visible and severe health problems as, for example, addiction to narcotics and psychoactive substances (Young, 1998). The problem is particularly serious, especially since the sources of virtual entertainment are being used by younger and younger people (especially children), who are not aware of their destructive impact on mental, physical, and social health. The definition of infoholic addiction is not currently standardized (Warzecha and Pawlak, 2017). One refers to the inability to function in society without direct or indirect contact with a telecommunication object. However, the problem can be much broader than just a difficulty with interpersonal interactions. In cyber addiction, the main causal object is the telecommunication device, which is most often the telephone (Chóliz, 2012). Often its connection to the user goes beyond the man–machine relationship, creating a kind of emotional bond. The cell phone becomes an object that provides experiences of various stimuli, including pleasure. As a result, phone addiction is characterized by the repetition of certain behaviors and strong devotion to the device, without which the user cannot do without. The psychological dependence is so strong that it often turns into a mental compulsion that requires repetition of the given activity (Chang and Law, 2008). The mechanism leads to an effect that relieves tension, psychological hunger, or improves wellbeing, and the addicted person is unable to stop the compulsive behavior on their own (Olszewska, 2013).

## Research Methodology

### Characteristics of the Study Group

The survey was conducted among players of urban 1,134 multimedia games (players of Pokemon GO, as the most popular application of this type) between March and June 2021 in Poland. For the final analyses, 990 correctly completed questionnaires (meeting the inclusion criteria described below and a filled-out form) were included.

There were 990 participants in the study, of which: 545 respondents were male (65.1%) and 445 female (34.9%). It was observed that the largest group of respondents were those aged 18–30 years—71.4% (average age 21 ± 2) of the respondents. The remaining group consisted of people aged above 30 years—28.6% (average 34 ± 1). In terms of education, the study group was as follows: 38.8% of the respondents were people with higher education, 35.7% with secondary education, 20.4% with primary education, and only 5.1% of the respondents had vocational education. Most of the respondents lived in big cities (more than 100 thousand inhabitants)—this answer was indicated by 61.2% of the respondents. A 54.1% of the respondents were not in a relationship (marital or civil partnership), of which 68.2% were men and 21.8% were women. Smaller towns and villages were inhabited by the rest of the surveyed group—respectively: 22,4% and 16,3%.

For the study, the profile of the gamer, i.e., the person who most often reaches for electronic entertainment in the form of urban multimedia games, was analyzed. This categorization was done by analyzing the most frequent results in the area of questions about sociodemographic data of the studied individual. Based on the observed trends, a risk threshold was determined, i.e., the characteristics that define the most frequent user of urban multimedia games. This leads to the identification of several characteristics of the general population of players. Based on the assumption that players of a similar type exhibit similar behavioral patterns, the collected information can be used to select a group of people among whom there is a higher risk (probability) of occurrence of certain characteristics.

### Research Tool

In the study, the author’s questionnaire was used, which included two standardized measurement scales in the abbreviated Polish version: Emotions and Sensations Related to Electronic Entertainment Questionnaire and Addiction to Electronic Forms of Entertainment Questionnaire. Both tools were developed by Kimberly Young (an expert on e-addiction) and.

tested for their psychometric properties, i.e., their accuracy and reliability allow for their use in the general population (Young, 1998). For the study, a pilot study was conducted using a questionnaire on a group of 30 people. A pilot study was conducted to validate the questionnaire and check the relevance and acceptability of the questions contained in it on a sample of people representing the default population of gamers. Cronbach’s α coefficient for the normalization sample was 0.89, which indicates a high reliability of the selected measurement scales. The pilot study allowed us to validate the questions contained in the questionnaire. Cronbach’s alpha for the relevant part of the study was estimated at 0.86, which is the same value as that obtained in the pilot sample.

The repeatability of responses was examined by comparing responses from the pilot sample and the general sample (30 pilot participants repeated the questionnaire in the general survey). The general survey took place one month after the piloting to avoid the freshness effect. To assess the reproducibility of the results obtained with the used questionnaire, the value of the parameter ϰ (Kappa) was calculated for each question in the questionnaire (results obtained in the pilot study and the general study)—for 61.3% of the questions a very good (ϰ ≥ 0,80) concordance of answers was obtained, while for 31.7% of the questions a good (0,79 ≥ ϰ ≥ 0,60) concordance of methods was obtained. Only for 7.0% of the questions in the analyzed questionnaire, the agreement between the results obtained in the baseline study and the retest was moderate (ϰ < 0,59). These were questions about the average time spent with the app/multimedia urban game and the amount of money spent on the game—with a tendency that on retesting respondents underestimated the values given previously. Scott’s coefficient was used to estimate the accuracy of classification in questions with a dichotomous structure (YES/NO). For questions: 1, 2, and 5 average relevance of π = 0.82—very good was obtained, and for questions: 3, 4, 6, and 8, it was π = 0.67—relevance good. A satisfactory level of relevance was obtained for question 7 (0.51). The methodology of the study was consulted from a psychometric point of view.

The interpretation of the result in the questionnaire of emotions and sensations related to electronic entertainment was based on taking the average of the obtained answers, which were arranged according to a five-point Likert scale (never, rarely, sometimes, often, and always) and measured the frequency of given emotions related to computer games and use of electronic forms of entertainment. In the electronic entertainment addiction questionnaire, the respondent answered 8 questions related to e-activities (YES/NO). An affirmative answer to at least 5 questions indicated an increased risk of computer game addiction—according to the clinical criteria proposed by the author of the method, Kimberly Young (1998).

The tool has been used before in research studies for Internet or mobile device addiction (Chang and Law, 2008; Chóliz, 2012; Warzecha and Pawlak, 2017). The results of all the studies indicate the high usability of the tool used.

### Inclusion Criteria

Data for the study were collected anonymously using the CAWI (Computer-Assisted Web Interview) method, the online questionnaire was distributed on forums and discussion groups dedicated to gamers (e.g., Pokemon GO facebook fanpages, Discord groups, and chat rooms for players). The criterion for inclusion in the study was the declaration of the use of mobile applications offering gameplay in augmented reality mode (mobile urban/outdoor games). In addition, only adults who consented to the study were included in the study, based on so-called spontaneous reporting. None of the respondents had previously received psychological counseling or psychiatric treatment.

Because the survey in question was conducted using the CAWI method, the sample selection was completely random (in accordance with the assumed criteria of inclusion and exclusion from the survey), but still, there was a certain risk of error, which could result in a random selection of survey participants, who were groups of people from close surroundings, who by default show similar behaviors and activities.

### Statistical Analyses

Statistica 13.0 software was used in the statistical processing of the data. Statistical tests were used to analyze the variables to make the statistical inference. For non-parametric characteristics and bivariate tables, the chi-square test was used. For other characteristics, the Kruskal-Wallis test and Mann-Witney U test were used. For statistical analyses, some data were recorded. The probability level adopted in the study was set at *p* ≤ 0.05.

## Results

After analyzing the data from the questions concerning gender, age, education, and place of residence, it is possible to identify the main group of recipients of urban multimedia games—hereinafter: the player profile. According to the above, they are usually men aged 18–30 with secondary education (more rarely higher), not in a relationship, living in cities with a population over 100 thousand—60.1% of players met these criteria.

In the next section of the questionnaire, respondents were asked, respectively, about the frequency of playing urban games and the change in their physical condition, mental condition, and interpersonal relations due to the start of the game. Respondents most often played multimedia urban games daily (84.7%) or every 2–3 days (11.2%). Only 4.1% of the sample of respondents used the app less frequently. As many as 70.4% of players had been playing since the game’s release in July 2016, 14.6% had been playing for about 2 years, and 10.0% for about a year. A single gaming session in the surveyed group was most often less than 2 h (52.6%), but there were some longer sessions. About 26.3% of the respondents played this type of game 2–3 h a day, and 14.7% played for more than 3 h. Over 90.0% of users take advantage of the micropayments available in the game, which allow them to purchase additional items and usability. On average, respondents spend more than PLN 50 per month on games—71.3%; less than PLN 50—28.7%.

In terms of physical condition, 64.3% of the respondents felt that their physical condition had improved as a result of playing urban multimedia games, while 33.7% felt that nothing had changed in this regard. Only 2% of the respondents’ physical activity worsened as a result of playing games of this type. This subjective evaluation of physical activity is reflected in the actual state (Table 1). The physical activity of the respondents increased, which is particularly evident in the increase of the time spent on active spending (from 30 to 60 min a day to more than an hour a day)—*p* < 0.05. This suggests that the fact of starting a game was an important motivator in the studied group to undertake physical activity.

**Table 1:** Level of physical activity by respondents.

**Physical activity (walking, running, cycling, etc.)**	**Before starting the game**	**After the start of the game**	**Statistics**
Lack of physical activity	10.3%	3.2%	*p* = 0.0001 *F* = 11.711
30–60 min per day	60.1%	22.3%	
More than 60 min per day	29.6%	74.5%	

Concerning mental health, most respondents reported no change (55.3%) and 23.7% reported increased self-confidence due to playing games. As many as 21, 0% perceived a worsening of their mental condition in connection with the game (negative feelings in the sphere of mental health are presented in Table 2). Negative feelings were found to be more frequent in those who met the conditions for the established gamer profile group (p < 0.05). Another variable was related to interpersonal relationships. Respondents were asked about how the game affected their interpersonal contacts. It was observed that in 36.7% of the respondents, these contacts improved. About 62.2% felt that nothing had changed in this matter, and 1.1% of the respondents noticed a worsening of their contact with the environment.

**Table 2:** Reported negative mental health feelings related to gaming by respondents.

**Perception**	**Total**	**Player Profile**	**Statistics**
Sadness/grief over losing	**10.3%**	**21.3%**	*p* = 0.0001 *F* = 15.621
Anger/nervousness/frustration about failing the game	**9.2%**	**15.6%**	
The general decrease in self-confidence	**7.3%**	**11.8%**	
Avoidance of social interaction (closing off to the environment)	**15.1%**	**19.2%**	

Table 3 presents data related to the advantages and disadvantages of playing urban multimedia games as indicated by the respondents. It should be noted that the most frequently indicated advantages were entertainment (killing boredom), improvement of physical activity, and desire to belong to a group. The disadvantages most frequently mentioned were the loss of time that could have been spent differently and the risk of addiction to this type of entertainment. In addition, among the disadvantages, respondents most often mentioned frustration and anger associated with the competitive aspect and the high risk of interpersonal misunderstandings that resulted from the formation of hermetic subgroups.

**Table 3:** Advantages and disadvantages of urban multimedia games according to respondents.

**Benefits**		**Faults**	
Entertainment aspect (stress relief)	64.6%	Loss of free time (addiction)	42.3%
Improving physical activity	23.2%	Frustration with competition	29.6%
Group membership	12.2%	Misunderstandings with people	28.1%

It was observed that 72.3% of the respondents were characterized by a certain risk of addiction to urban multimedia games (in the screening questionnaire on addiction to electronic forms of entertainment they marked at least 5 affirmative answers in the examined subscales), and 93.1% of people with an increased risk of addiction were those with the player profile shown in the first part of the description of the results. At this point, a statistical dependence was demonstrated—persons displaying the above-mentioned characteristics (male sex, age 18–30, single status, min. Secondary education, and place of residence in a city of more than 100,000 inhabitants) more often tend to become addicted to electronic forms of entertainment (*p* < 0.05). Moreover, it was observed that in the studied group, the frequency of negative experiences related to electronic entertainment may indicate their problematic use of the virtual medium. Detailed data are presented in Tables 4, 5. The interpretation of the result is explained in the Research methodology section.

**Table 4:** Questionnaire of addiction to electronic forms of entertainment.

Subscale (questions)	Total		Player profile		Statistics
	Affirmative answer “Yes”	Answer denying “No”	Affirmative answer “Yes”	Answer denying “No”	
Do you feel					*p* = 0.0001 *F* = 13.281
preoccupied with the					
game to the point					
that you constantly think about the	51.2%	48.8%	**85.3%**	14.7%	
sessions you have had					
and/or cannot wait for					
the next sessions?					
Do you feel the need					
to increase the					
amount of time you					
spend playing the	64.1%	35.9%	**78.2%**	21.8%	
game to get more					
satisfaction. to feel					
more satisfaction?					
Have you made					
unsuccessful					
attempts to control,	49.2%	50.8%	**69.3%**	30.7%	
Limit, or stop your use					
of the game?					
Do you feel anxious,					
depressed or irritable					
when you have tried	**52.3%**	47.7%	50.8%	49.2%	
to limit or stop your					
use of the game?					
Do you find yourself,					
spending more time in the game than you	**83.4%**	16.6%	71.9%	28.1%	
originally planned?					
Have you ever risked losing a loved one, important relationships, job, education, or career because of spending too much time gaming?	65.0%	35.0%	**81.0%**	19.0%	
Have you ever lied to your loved ones or someone else to hide your excessive interest in gaming?	23.8%	**76.2%**	46.1%	53.9%	
Have you used the game to escape from problems or to avoid unpleasant feelings (e.g., feelings of helplessness guilt, anxiety, or depression)?	43.0%	57.0%	**71.0%**	29.0%	
Increased risk of addiction (min. 5 affirmative responses)	**72.3%**		**93.1%**		
No risk of addiction (0–4 affirmative responses)	**27.7%**		**6.9%**		

*Bold values are the highest values in a given section.*

**Table 5:** Emotions and experiences associated with electronic entertainment according to respondents (*N* = 990).

Subscale (questions)	Never	Rarely	Sometimes	Often	Always
Have you ever spent more time in a game than you intended?	5.1%	2.5%	**64.6%**	5.1%	22.7%
Do you find yourself putting off chores to have more time to play?	6.6%	13.6%	28.3%	**35.4%**	16.2%
Do you happen to interact with other players?	0.5%	1.5%	2.5%	**90.4%**	5.1%
Does your entourage complain about you spending too much time gaming?	3.0%	5.6%	1.5%	**87.4%**	2.5%
Is your time in the game negatively impacting your work?	4.5%	**51.5%**	33.8%	5.1%	5.1%
In the first place, do you happen to check your game progress before you do something useful?	7.6%	7.6%	**62.1%**	1.0%	21.7%
Do you see a negative impact of the game on your performance?	3.5%	15.2%	**41.4%**	11.6%	28.3%
Do you often drive away negative thoughts about your life by comforting yourself with the thought of the game?	3.0%	1.0%	**38.4%**	25.3%	32.3%
Do you happen to get excited at the very thought of turning on a game?	6.1%	19.7%	7.1%	**54.5%**	12.6%
Do you sometimes fear that life without games, would be boring, empty and devoid of charm?	4.0%	4.0%	40.4%	**45.5%**	6.1%
Do you find yourself getting annoyed, raising your voice, or not responding politely when someone bothers you while you are playing?	8.6%	7.6%	**60.6%**	15.7%	7.6%
Do you ever find yourself thinking intensely about a game when you are not currently playing?	4.5%	4.5%	**76.8%**	7.1%	7.6%
Do you happen to say: “one more moment and I’m off”?	0.0%	0.0%	16.2%	**72.2%**	11.6%
Have you ever made unsuccessful attempts to reduce your time spent in a game?	5.1%	2.5%	**64.6%**	5.1%	22.7%
Do you conceal the amount of time you spend in the game from others?	6.6%	13.6%	28.3%	**35.4%**	16.2%
Do you find yourself irritable and in a bad mood when you are not playing, and in a good mood when you are?	0.5%	1.5%	2.5%	**90.4%**	5.1%

*Bold values are the highest values in a given section.*

## Discussion

In recent years, there has been a significant increase in interest in electronic entertainment, especially games using augmented reality. Moreover, the easy availability of such entertainment on mobile devices makes playing urban games fashionable in literally every age group. Considering the inclusion criterion of the self-reported study, the most frequent gamers were adult males.

Based on the research, the profile of the players was estimated. They are most often men aged 18–30 with secondary education (more rarely higher), not in a relationship, living in cities with a population over 100,000—60.1% of players met these criteria. The results of our study are the same as the results of other studies, for example, the study of Laor et al. (2021b) also showed that the most frequent players of urban games are adult males (67%, mean age 25.05 years).

In a study conducted in the US (2016), the effects of urban games on physical activity levels were investigated using wearable pedometers. The study was conducted among 792 Pokemon GO players and found that playing Pokemon GO led to a significant increase in distance walked, by an average of 1,473 steps per day, over 30 days (Althoff et al., 2016). The same trend was observed in a similar study, where Pokemon GO users reported approximately 950 additional steps per day within the first week of installing the game. However, the number of daily steps dropped significantly in the sixth week after installing the game (Howe et al., 2016). A study of college students also found that Pokemon GO can be an effective tool for increasing physical activity levels (Barkley et al., 2017; Marquet et al., 2017, 2018). In contrast, a cross-sectional study of Hong Kong university students found no significant difference in physical activity levels between Pokemon GO players and non-players within 4 weeks after the game’s release (Wong, 2017). In our study, according to the subjective assessment of physical activity, the subjects observed an increase in physical activity since starting to play this type of game (64.3%).

Most previous studies have focused on the effects of Pokemon GO on physical activity levels within the first 4–6 weeks of playing (Althoff et al., 2016; Howe et al., 2016; Wong, 2017). While other studies have shown an increase in physical activity within the first few weeks of starting the game (Althoff et al., 2016; Howe et al., 2016), some have shown that gaming does not increase activity in young or older adults (Rasche et al., 2017; Wong, 2017). Results from a 3-month follow-up showed that the game did not change the physical activity levels of participants in the study group, and the number of players decreased significantly. Studies show that the app alone is not a good method to increase physical activity, but its combination with social activities can have a much better impact (Althoff et al., 2016; Wong, 2017). The results of the author’s study show that the majority of app users use the app daily (84.7%), and mostly a single session in the game lasts up to 2 h.

Among the main reasons for playing Pokemon GO in study participants were no increase in physical activity—only a quarter of participants played the game to improve physical fitness. Therefore, the increase in physical activity levels observed in some studies (Althoff et al., 2016; Howe et al., 2016) may be a positive side effect of the game (McCartney, 2016). Although players were required to travel to public places to continue the game (Anderson et al., 2016: Ayers et al., 2016), some players traveled by vehicle instead of walking. This may explain the lack of correlation between physical activity level and time spent playing the game. Furthermore, although a significant decrease in sedentary resting time was found among Pokemon GO players, it was not directly correlated with time spent playing the game. This may be an indirect effect of the game causing players to be outdoors (Wong, 2017). In our study, we observed a positive effect of the game on mental state and interpersonal communication. The improvement of mental state with self-confidence was indicated by 33.7% of the respondents, while 62.2% of the respondents met many new friends. In addition, among the main 3 advantages of playing multimedia urban games, respondents indicated belonging to a group (12.2%). Nevertheless, they indicated frustration related to competition (29.6%) and misunderstandings with people (29.1%) as the main disadvantages of the game.

In addition, many scientific publications predict negative effects of urban gaming on health, especially mental health—addiction and loss of control over one’s life (Vallerand et al., 2003; Przybylski et al., 2009; Orosz et al., 2016). According to research and current motivational correlates, gamers may be less motivated to play to improve their mental or physical wellbeing. In the study, passion for gaming was strongly associated with motives of escapism and boredom (Wattanapisit et al., 2018). Consistent with previous results, playing online games to escape real-life problems may lead to problematic use (Laor, 2020). Therefore, gamers who engage in urban games to escape from reality may be at risk of developing problematic gaming behaviors. Goal achievement and competition were also expected to be associated with the desire to play, similar to more general findings (Király et al., 2015) regarding online gaming. The association of boredom with the willingness to play may be explained by rigid, unsatisfying engagement in the activity (Vallerand et al., 2003). Furthermore, imaginative imagery was found to be positively related to willingness to play. Based on the positive pattern of the relationship between passion, achievement, escapism, and fantasy, we can speculate that fantasy imaginings can also be interpreted, as a creative internal form of escapism (Orosz et al., 2016). In our study using the questionnaire of emotions and sensations related to electronic entertainment, it was shown that 72.3% of the respondents are characterized by the risk of addiction to urban multimedia games. In addition, the frequency of negative sensations associated with electronic entertainment may indicate their addiction to the virtual world. Respondents mostly (42.3%) indicated the possibility of addiction (loss of free time) as the main disadvantage of the game.

The available literature on modern media addiction consistently emphasizes that individuals addicted to video games, including mobile apps, are more likely to report symptoms of anxiety and depressive disorders. Media addiction is associated with poorer emotional health (Bruchas et al., 2011). For example, Whang et al. (2003) observed a significant association between the degree of game and Internet addiction and loneliness and depression. Adolescents with high levels of Internet use exhibited more psychopathology as revealed by the Brief Symptoms Inventory (BSI) compared to those with low levels of online game use (Yen et al., 2008). In addition, one study found that young adults addicted to video games showed a greater predisposition to depression and anxiety and felt more socially isolated. The link between mobile game addiction and mental health may be due to social isolation resulting from spending too much time playing games, which in turn leads to poorer mental wellbeing (Kraut et al., 1998).

### Strengths and Limitations

In the strengths of the conducted research, it should be emphasized that despite the popularity of such forms of entertainment, there are still few scientific studies that treat mobile urban games in terms of the possibility of addiction and negative impact on various spheres of life, including mental health. Usually, studies include the factor of physical activity, which was proved by the conducted discussion of the results. Bibliometric analysis of PubMed indicates that currently 8,339 papers have been cataloged on the topic of multimedia entertainment in total, of which 1,010 are related to gaming on mobile devices, and only 92 are related to mobile urban games, of which 71 are research papers directly related to health behaviors (prevention of overweight and obesity, physical activity), 6 papers are review papers on similar topics, and only 15 papers deal with aspects of mental health, including addiction to this type of entertainment (as of 23.01.2022).

The survey used material collected from 990 respondents, which is a representative group of players of this type of entertainment. One of the limitations of the conducted research is the method of data collection, which was carried out in the CAWI system, i.e., using an Internet form. This type of research is often accused of lower reliability of the answers given, although it should be emphasized that the specific nature of the group studied (age and the use of electronic devices) allowed for easy access using the above-mentioned Internet tools. In addition, research shows that the use of the Internet in conducting surveys allows the respondent to give more thoughtful answers (Alessi and James, 2010). Moreover, in the prepared questionnaire, in addition to its questions, psychometric tools were used, which were previously used in scientific research (Krajewska-Kułak et al., 2010; Wang et al., 2019).

The study chose not to include a control group of non-gamers, as the type of questions asked would not allow for comparisons between such groups. Instead, it was decided to make a comparison between the general group of gamers and a group of people who fit certain characteristics into the gamer profile established in the study.

Future studies should include young people (school-aged children and adolescents), as many studies show that such individuals are more likely to develop addictions related to widely available electronic devices and software (Whang et al., 2003; Laor, 2020). Despite this, the study described in this paper did not choose to do so because it is difficult to recruit such a group *via* the Internet (forums and newsgroups), and such people are often characterized by short-term interest and do not constitute a group of regular players of urban games (Yen et al., 2008).

## Conclusion

Based on the research conducted and the review of the scientific literature, it can be concluded that users of urban multimedia games were usually adult males living in large cities. It was also shown that the defined profile of the gamer was characterized by a higher risk of addiction to electronic forms of entertainment. In addition, it was observed that the respondents mostly played multimedia urban games daily in a maximum of two-hour sessions. In their subjective assessment, the game did not affect their psychosocial functioning and they even observed an increase in physical activity. Based on the tools used, it can be concluded that the study group was characterized by a certain risk of addiction to urban multimedia games. This phenomenon should be controlled, and users should be educated on the hygienic use of electronic devices and the limit of time spent in the virtual world.

In conclusion, it can be said that the aim of the study was fulfilled and the research question posed in the study was answered. As previously described further research should focus on the risk of addiction to modern media, including urban multimedia games in younger age groups.

## Data Availability Statement

The original contributions presented in the study are included in the article/supplementary material, further inquiries can be directed to the corresponding author.

## Ethics Statement

The study involving human subjects was reviewed and approved by the Bioethics Committee of the Silesian Medical University in Katowice, Poland. Study participants gave written informed consent to participate in this study. All study participants were informed of the purpose of the study, its anonymity, and were asked to accept the data sharing policy. Information about informed and voluntary participation in the study was at the beginning of the questionnaire. The study was approved by the Bioethics Committee of the Medical University of Silesia in Katowice in light of the Act on Medical and Dental Professions of 5 December 1996, which includes a definition of medical experimentation.

## Author Contributions

MG and ŁO: conceptualization, investigation, and original draft preparation. MG and KK-K: methodology and data curation. AB-D: review and editing. KS-N: supervision. MG: project administration. All authors contributed to the article and approved the submitted version.

## Conflict of Interest

The authors declare that the research was conducted in the absence of any commercial or financial relationships that could be construed as a potential conflict of interest.

## Publisher’s Note

All claims expressed in this article are solely those of the authors and do not necessarily represent those of their affiliated organizations, or those of the publisher, the editors and the reviewers. Any product that may be evaluated in this article, or claim that may be made by its manufacturer, is not guaranteed or endorsed by the publisher.
